# Gaining Insight into Mitochondrial Genetic Variation and Downstream Pathophysiology: What Can i(PSCs) Do?

**DOI:** 10.3390/genes12111668

**Published:** 2021-10-22

**Authors:** Jesse D. Moreira, Deepa M. Gopal, Darrell N. Kotton, Jessica L. Fetterman

**Affiliations:** 1 Evans Department of Medicine and the Whitaker Cardiovascular Institute, Boston University School of Medicine, Boston, MA 02118, USA; jessedm@bu.edu (J.D.M.); dmgopal@bu.edu (D.M.G.); 2 Cardiovascular Medicine Section, Department of Medicine, Boston University School of Medicine, Boston, MA 02118, USA; 3 Boston Medical Center, Center for Regenerative Medicine of Boston University, Boston, MA 02118, USA; dkotton@bu.edu; 4 The Pulmonary Center, Department of Medicine, Boston University School of Medicine, Boston, MA 02118, USA

**Keywords:** mitochondria, oxidative phosphorylation, induced pluripotent stem cells, heteroplasmy

## Abstract

Mitochondria are specialized organelles involved in energy production that have retained their own genome throughout evolutionary history. The mitochondrial genome (mtDNA) is maternally inherited and requires coordinated regulation with nuclear genes to produce functional enzyme complexes that drive energy production. Each mitochondrion contains 5–10 copies of mtDNA and consequently, each cell has several hundreds to thousands of mtDNAs. Due to the presence of multiple copies of mtDNA in a mitochondrion, mtDNAs with different variants may co-exist, a condition called heteroplasmy. Heteroplasmic variants can be clonally expanded, even in post-mitotic cells, as replication of mtDNA is not tied to the cell-division cycle. Heteroplasmic variants can also segregate during germ cell formation, underlying the inheritance of some mitochondrial mutations. Moreover, the uneven segregation of heteroplasmic variants is thought to underlie the heterogeneity of mitochondrial variation across adult tissues and resultant differences in the clinical presentation of mitochondrial disease. Until recently, however, the mechanisms mediating the relation between mitochondrial genetic variation and disease remained a mystery, largely due to difficulties in modeling human mitochondrial genetic variation and diseases. The advent of induced pluripotent stem cells (iPSCs) and targeted gene editing of the nuclear, and more recently mitochondrial, genomes now provides the ability to dissect how genetic variation in mitochondrial genes alter cellular function across a variety of human tissue types. This review will examine the origins of mitochondrial heteroplasmic variation and propagation, and the tools used to model mitochondrial genetic diseases. Additionally, we discuss how iPSC technologies represent an opportunity to advance our understanding of human mitochondrial genetics in disease.

## 1. Introduction

Mitochondria are double membrane-bound organelles residing ubiquitously in the cells of eukaryotic organisms. Mitochondria are multifaceted organelles that participate in many cellular activities, the most critical of which is the production of molecular energy in the form of adenosine triphosphate (ATP). Mitochondrial ATP production is largely driven by oxidative phosphorylation (OXPHOS). The reducing equivalents nicotinamide adenine dinucleotide (NADH) and flavin adenine dinucleotide (FADH_2_) donate electrons through a series of electron transport chain enzyme complexes to establish a proton gradient across the inner mitochondrial membrane. The final OXPHOS complex, ATP synthase, harnesses the electrochemical gradient to produce cellular ATP [1,2].

Highlighting the co-evolution of mitochondria and eukaryotic cells, the OXPHOS enzyme subunits are encoded by both the mitochondrial DNA (mtDNA) and nuclear DNA (nDNA). Although the nDNA encodes the bulk of the OXPHOS complex subunits, the mtDNA encodes key catalytic subunits of OXPHOS complexes I, and III-V. Only complex II is entirely nDNA-encoded. The nDNA-encoded OXPHOS subunits play key roles in the assembly, stability, and regulation of the OXPHOS complexes. Further, the nDNA encodes mitochondrial chaperones, import machinery, and proteins involved in mtDNA replication.

Mitochondrial respiratory chain disorders, often with diverse clinical presentations, affect an estimated 1 in 4300 individuals [3,4]. Pathogenic mutations may arise in either genome and result in defective OXPHOS, which drives alterations in upstream metabolic pathways. The age and phenotypic presentation of mitochondrial diseases varies significantly across individuals, even within the same family, creating challenges in diagnosis and disease management. Currently, no therapeutic strategies for the treatment of mitochondrial genetic disease are FDA-approved [5].

Multiple copies of the mtDNA exist within a cell. A mitochondrion or cell can be either homoplasmic or heteroplasmic. Mitochondria or cells that contain mtDNA’s that are identical are considered homoplasmic, whereas mitochondria or cells containing mtDNA’s with different genetic variants are considered heteroplasmic. Although homoplasmic mtDNA mutations can cause mitochondrial disease, most frequently, phenotypic mitochondrial disease is due to heteroplasmic mutations. Patients with mitochondrial disease often have different distributions of the pathogenic heteroplasmic variant across tissues, which likely drives the phenotypic presentation. Further, the number of mtDNA copies with the heteroplasmic variant varies widely across mitochondria and cells, as well as varying across tissue types.

Advancing age is correlated with the accumulation of heteroplasmic variants [6], which are more likely to alter the function of the OXPHOS complex. Consequently, it is thought that mitochondrial dysfunction, resulting from increased mtDNA heteroplasmy, may underlie some of the distinctive pathological features of aging and age-related diseases such as cardiovascular disease and dementia. As such, studying how mitochondrial variants impact mitochondrial function across the lifespan may reveal novel ways to treat age-related illnesses, saving millions in medical care costs and extending lifespans.

The advent of next generation and ultra-deep sequencing has increased our ability to detect low level heteroplasmic mtDNA variants and comprehensively evaluate mitochondrial genetic variation across both genomes simultaneously. Delineating pathogenic variants from benign polymorphisms is difficult due to the significant variation in the mtDNA and limitations in the ability to validate variants of unknown significance or suspected to be pathogenic. Access to the affected tissues in the setting of mitochondrial disease is often not feasible. Consequently, the diagnosis of mitochondrial diseases is often restricted to genetic and biochemical evaluation of blood samples, dermal fibroblasts, or skeletal muscle biopsies. Modeling human mitochondrial disease has been challenging due to substantial interspecies mitochondrial genetic variation, often precluding the use of animal models.

Advances in human induced pluripotent stem cell (iPSC) and clustered regularly interspaced short palindromic repeats (CRISPR-Cas9) gene-editing technologies provide new opportunities for advancing our understanding of the underlying mechanisms of mitochondrial disease. Herein, we provide an overview of the challenges and barriers in studying the contribution of mitochondrial genetic variants to mitochondrial and age-related diseases. Importantly, we discuss that iPSCs can undergo directed differentiation towards any adult cell type, including cardiomyocytes and neurons. Through the use of gene-editing technologies, iPSC-derived cells facilitate the exploration of the mechanisms of mitochondrial genetic disease, allowing for the identification of causal relationships between mitochondrial genetic variants and cellular phenotype to drive the identification of novel therapeutics.

## 2. The Mitochondrial Genome

### 2.1. Structure and Maintenance of the mtDNA

mtDNA is a circular, double-stranded chromosome of approximately 16,569 base pairs localized to the mitochondrial matrix side of the inner mitochondrial membrane. Each mitochondrion is thought to contain 5–10 copies of the mtDNA and depending on the cell type, between 500 and 6000 copies of the mtDNA exist per cell [6]. The mtDNA consists of a noncoding origin region, called the D-loop, 13 OXPHOS peptide-encoding genes, 22 transfer RNAs (tRNA) and 2 ribosomal RNA (rRNA) regions for translation of the 13 peptide-encoding genes [7]. Importantly, the bulk of the catalytic subunits of the OXPHOS enzymes are encoded by the mtDNA.

Unlike nDNA, mtDNA is not bound by histones. Instead, mtDNA is folded and maintained via various DNA binding proteins, including single-stranded binding proteins and transcription factors. Together, the various binding proteins and condensed mtDNA are termed nucleoids. The most abundant nucleoid protein is the mitochondrial transcription factor A (TFAM) [8], which is ubiquitously associated with each mtDNA molecule at a ratio of 1000:1, and has a sequence-non-specific binding capability that allows it to regulate the topology of mtDNA [9,10]. Whether the other nucleoid-associated proteins compose the nucleoid or are simply transiently associated has yet to be determined. Despite the formation of nucleoids, however, mtDNA is not as tightly packaged as nDNA, which may contribute to the higher mutation rate of mtDNA [11,12,13], along with its close proximity to the electron transport chain, a source of oxidants. 

The mtDNA is replicated by the DNA polymerase γ (POLγ), for which there is no substitute [14]. The helicase TWINKLE begins replication at the heavy strand origin, working directly ahead of POLγ. Single-stranded binding proteins cover the single stranded parent heavy strand to prevent secondary structure formation and aberrant transcription while the new heavy strand is synthesized. Following passage of the light strand origin, about two thirds of the way through heavy strand synthesis, the newly freed light strand origin forms a stem loop and is associated with a primer, which after about 25 nucleotides is replaced by POLγ, initiating transcription of a new light strand [7,15,16]. Importantly, errors of replication are considered a major source of spontaneously arising mitochondrial genetic variation and can be a causal factor in the accumulation of heteroplasmic mitochondrial populations [17,18]. As such, errors of replication are important considerations in mitochondrial diseases.

### 2.2. Sources and Segregation of mtDNA Heteroplasmic Variants

Emerging evidence suggests heteroplasmy, a condition in which alternative alleles are present in a single cell’s mitochondrial genomes, is widely present across the mitochondrial genome in low amounts (~1–2%) in most, if not all, humans [19,20]. Much like the nDNA, an alternative mtDNA allele may change the overall phenotypic presentation associated with a gene. At such low levels, mtDNA variants are thought to be harmless. However, due to genetic bottlenecking, uneven segregation of mitochondrial populations, relaxed mtDNA replication, and both the positive and negative selection of pathological mtDNA variants by environmental pressures, high levels of heteroplasmic mtDNA may accumulate in a given tissue, resulting in disease [6,19,21,22].

**Figure 1:**
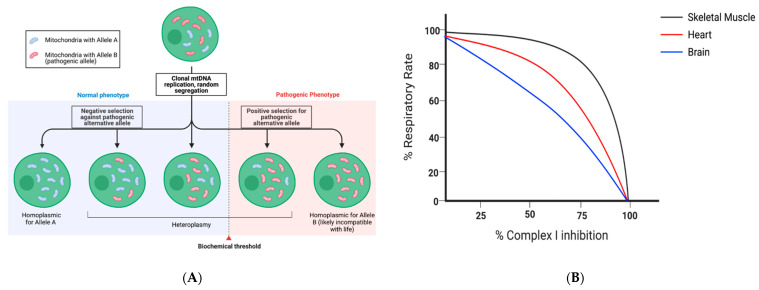
(**A**): Mitochondrial segregation can result in a spectrum of progeny, which harbor a range of mtDNA variants dependent upon random segregation and/or positive and negative environmental selective pressures. Notably, time may influence the segregation process, including biological age. (**B**): While the biochemical threshold shown in 1A is static, the true biochemical threshold for heteroplasmic variation functionally affecting the tissue varies by tissue type. Data adapted from [23]. Figures created with biorender.com (accessed on 10 October 2021).

Heteroplasmic mtDNA variants may initially arise as a result of direct base addition error in DNA replication. Errors of replication, and resultant heteroplasmy, may not necessarily be problematic in low quantities. For example, if 1% of the genes encoding for an OXPHOS complex I subunit vary in sequence, but the other 99% of mtDNAs contain the reference allele, the phenotype may be nonexistent or subclinical. However, with higher levels of heteroplasmy, a biochemical disruption in OXPHOS may occur, resulting in phenotypic changes in cell function and ultimately, the onset of disease [23] (Figure 1A). The exact percentage of mtDNA copies that must possess a specific heteroplasmic variant in order to result in a pathologic phenotype, however, will range by tissue, gene, and position (Figure 1B). Together, the uneven tissue distribution and uneven percentage of the genes possessing the heteroplasmic variant highlight the difficulty in determining the exact contribution of heteroplasmic variants to disease phenotypes.

A major mechanism contributing to the generational persistence of heteroplasmy is genetic bottlenecking. Bottlenecking is the phenomenon where the total mtDNA quantity in the developing embryo is decreased due, in part, to lower oxygen tensions prior to blastocyst implantation [21]. During development in females, germ line progenitors undergo the mitochondrial genetic bottleneck, which determines the segregation of specific mtDNA copies in the germ line progenitors into the various forming germ cells. mtDNA segregation is intended to decrease pathogenic mitochondrial heteroplasmic variation to prevent the transmission of pathogenic variants to offspring. The mitochondrial genetic bottleneck determines the mtDNA sequences that will give rise to an organism’s somatic cells, as well as the individual’s progeny, as the female germ line progenitor cells will provide mitochondria to the future offspring. As such, if novel mutations arise during the genetic bottleneck, or if there are selective pressures that result in a failure to eliminate heteroplasmic variants, the future progeny may inherit heteroplasmic mtDNA. Recent evidence has shown that maternal inheritance plays a significant role in the acquisition of heteroplasmic variation [20,24,25]. Ultimately, the mechanisms mediating mtDNA segregation into germ cells and the selection processes that maintain homoplasmic mtDNA sequences during this segregation are critical points for the introduction of heteroplasmic variation, and research is needed to identify the mechanisms mediating this process.

### 2.3. Selective Pressures and Heteroplasmic Variants

The selective pressures operating on mitochondrial heteroplasmic variants are complex. Both positive and negative selective pressures influence the segregation of heteroplasmic mtDNA variants and alter their presence and levels in cellular populations or tissues [21,24]. The mitochondrial genetic bottleneck, for example, is subject to environmental selective pressures. Under low oxygen tensions, germ-line progenitor-like cells have been shown to have higher levels of heteroplasmic variance during events representative of the mitochondrial genetic bottleneck [21]. In contrast, future somatic-like cells displayed a low oxygen tension-dependent decrease in heteroplasmic segregation [21]. The difference in positive and negative selection acting on heteroplasmic variants under a simulated genetic bottleneck demonstrates the highly context-dependent influence of environmental cues on mtDNA heteroplasmy in discrete cell types.

Following the inheritance of heteroplasmic variants into a germ cell, it is currently unknown if a mechanism governs segregation of mtDNA through the developing embryo or if the process is random [19,26]. If it is random, as it seems from computational models, this helps explain the uneven proportion of heteroplasmic variants across tissues in any given disease, even between individual siblings carrying the same pathogenic mutation. Random segregation, for example, has been shown to occur in the presence of non-pathogenic mtDNA variants, with very few tissues remaining unsegregated [27]. However, non-random patterns of segregation have also been shown, although the mechanism for non-random selection remains unclear [28].

One of the challenges in the diagnosis of mitochondrial disease is that the pathogenic variant may not be present in blood cells as a result of negative selective pressure [29,30]. As blood cells have a fairly turnover, it is believed that they are able to undergo selection against pathogenic variants. Consequently, skeletal muscle biopsy is often performed when mitochondrial disease is suspected as skeletal muscle is a relatively accessible post-mitotic tissue, thought to be representative of other post-mitotic cell types [31]. Further, even different blood cell types differ in the presence and variant allele frequency of heteroplasmic variants [32]. In single cell sequencing of blood samples from three unrelated patients with mitochondrial encephalopathy, lactic acidosis, and stroke-like episodes (MELAS), the heteroplasmic levels of the pathogenic m.3243A > G variant varied across the peripheral blood mononuclear cells with lymphocytes, particularly CD4^+^ and CD8^+^ T cells, having much lower levels of the heteroplasmic variant than myeloid-lineage cells.

Lastly, environmental exposures also interact with the mtDNA and modulate the penetrance of mitochondrial disease. In Leber’s hereditary optic neuropathy, cigarette smoking triggers the onset of disease due to a decline in mtDNA copy number and impaired mitochondrial biogenesis [33,34,35]. Of note, Leber’s hereditary optic neuropathy penetrance is lower in females, likely due to the role of estrogens in maintaining higher mtDNA copy numbers and mitochondrial mass in females compared to males. Additionally, many mitochondrial disorders disproportionately affect males, as it has been hypothesized that mitochondrial variation has evolved to not harm, or reduce the biological fitness of, females [36].

The presence and levels of heteroplasmic variants likely change over time in a cell type dependent manner, along with environmental interactions, which can be challenging to study in humans. To date, our understanding of the pressures acting on heteroplasmic variation and distribution within the body are largely the result of studying individuals with mitochondrial disease, in which the causal variant is a heteroplasmic mtDNA variant. As such, we still have much to understand regarding the effect of variants of unknown significance, or how variants that present exclusively in tissues that are difficult to obtain, such as brain or heart biopsies, hindering research pace, drive disease. We are just beginning to dissect how selective pressures modulate heteroplasmic variant segregation and persistence in human disease.

## 3. Mitochondrial Physiology: Genes to Respiration and Metabolism

### 3.1. OXPHOS Substrate Input and Feedback Regulation 

Most cellular ATP is generated through OXPHOS. The reducing equivalents that drive OXPHOS are derived from a variety of sources. In eukaryotic cells, NADH and FADH_2_ are the primary products of the Krebs cycle, with fatty acid oxidation and anaerobic glycolysis supplying additional reducing equivalents [37,38]. Reducing equivalents donate electrons to the OXPHOS complexes, entering the electron transport chain at complexes I (NADH) and II (FADH_2_). The flow of electrons through the OXPHOS complexes facilitates the transfer of protons across the mitochondrial inner membrane to the intermembrane space, setting up the proton motive force that drives the rotor-like enzyme ATP synthase. Turning of the rotor of ATP synthase (complex V) results in ATP generation from ADP and inorganic phosphate.

**Figure 2:**
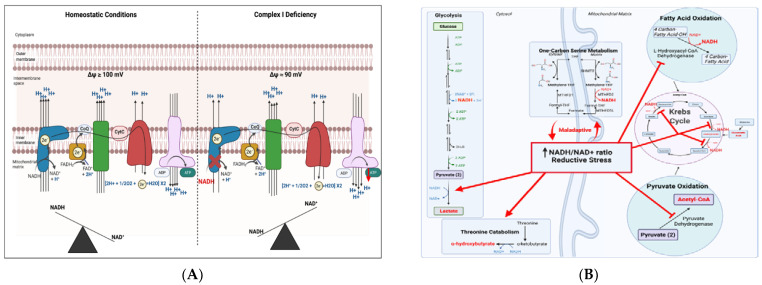
(**A**) Mechanisms of Complex I deficiency. Impairment of complex I results in fewer electrons transferred from NADH into the electron transport chain, and consequently, fewer protons pumped across the inner mitochondrial membrane, resulting in a lower membrane potential (ΔΨ). The lower membrane potential results in a decreased proton motive force with less ATP generation. Complex I shown in dark blue; II shown in yellow; III shown in green; IV shown in dark red; V shown in pink; CoQ 10 shown I light blue; Cytochrome C shown in light red. (**B**) Illustration of the effects of reductive stress, such as occurs in Complex I deficiency, on various metabolic pathways. Highlighted in red are molecules demonstrated to be increased in abundance in reductive stress. Figure created with biorender.com (accessed on 10 October 2021).

Of note, the reducing equivalent NADH, used as a substrate by complex I, is involved in mediating end-product inhibition of pyruvate dehydrogenase, the main driver of glycolysis-derived acetyl CoA production. As such, an increase in NADH/NAD+ results in suppression of further NADH production under homeostatic conditions (Figure 2). High NADH/NAD+ ratios also inhibit the beta-oxidation of fatty acids, the other major source of cellular acetyl CoA [39]. Typical of homeostatic processes, this system uses a simple evolutionary mechanism for the inhibition of redundant enzymatic activity: end-product inhibition. After end-product inhibition, the re-generation of NAD+ then releases enzyme inhibition resulting in a resumption of activity of fatty acid oxidation and glycolysis. Hence, product inhibition by NADH is a major source of regulation of reducing equivalent production across multiple metabolic pathways and is critical to intracellular metabolic balance.

### 3.2. Effects of Heteroplasmic Variance on OXPHOS Regulation 

MtDNA heteroplasmic variants in the OXPHOS-encoding genes are more likely to be missense mutations, resulting in an amino acid change, and hence, more likely to alter the activity of the OXPHOS complex [6]. Mutations affecting complex I (NADH:ubiquinone oxidoreductase) are among the most well-studied, causing mitochondrial complex I deficiency disorders. Impairments in complex I result in the accumulation of NADH. Excessive NADH/NAD+ ratios result in a chain of events disrupting cellular homeostasis. Excessive and prolonged end-product inhibition of the Krebs cycle by elevated NADH, for example, results in several changes. First, end-product inhibition causes the suppression of several other important metabolic intermediates (succinate, malate, etc.) and excessive shunting of the built-up pyruvate towards lactate production [40,41]. Excessive NADH levels also inhibit fatty acid oxidation. The reductive stress as a result of elevated NADH/NAD+ alters cellular homeostasis and affects downstream signaling pathways, influencing epigenetic regulatory mechanisms and resultant transcriptional states [42]. 

Enhanced one-carbon metabolism is an emerging maladaptive upstream mechanism contributing to NADH-reductive stress. Following pharmacologic inhibition or genetic mutation of complex I, excessive NADH levels are, in part, attributable to an upregulation of folate cycle-dependent one-carbon serine metabolism [43,44]. Even when high NADH/NAD+ ratios would theoretically inhibit further NADH production to facilitate normalization of NADH levels, one-carbon metabolism continues to drive NADH production due to the absence of end-product inhibition, ultimately contributing to and sustaining the reductive stress. In a murine model of complex I deficiency, sustained serine catabolism led to progressive mitochondrial disease featuring spasticity [44]. In contrast, pharmacological inhibition of serine metabolism in the complex I deficient mice rescued the reductive stress-dependent pathology, including normalizing cell growth, and reducing whole-animal spasticity. Hence, one-carbon metabolism during OXPHOS failure is like a contributor to NADH-dependent reductive stress (Figure 2B).

Experiments delineating the cascade of metabolic aberrations following mtDNA-related OXPHOS deficiency show how clearly a single mutation can affect cellular homeostasis. Metabolites reflective of reductive stress (α-hydroxybutyrate, C2:0-carnitine, C3:0-carnitine, acylcarnitines) are elevated in plasma from patients with mitochondrial disease compared to well-matched referents [3], indicating the potential utility of metabolomic approaches for the diagnosis of mitochondrial disorders [3].While hundreds of mtDNA variants are suggested causal mutations in human disease phenotypes, only approximately 80 variants have been confirmed as causal while over 500 variants are still awaiting confirmation [45]. Significant work is needed to characterize the effects of heteroplasmic mtDNA variants of unknown significance on human metabolism to identify novel diagnostic and therapeutic targets.

## 4. Modeling Mitochondrial Disease

A number of case-studies have described mitochondrial diseases, however, consensus on the diagnosis and treatment of mitochondrial disease continues to be elusive. The Mitochondrial Medicine Society published guidelines in 2015 [31], but importantly, no FDA approved therapies are available for the treatment of mitochondrial disease [46]. Current treatment recommendations are supportive in nature. The lack of a consensus on the management of mitochondrial disease is due to several factors. First, mitochondrial diseases are highly heterogeneous, with the phenotypic presentation largely dependent upon the mutational presence and burden within tissues. Second, the biochemical basis of mitochondrial diseases in cells or tissue from humans is poorly characterized. A key challenge in improving the diagnosis and treatment of mitochondrial disease is the rarity of the disease, making large-scale clinical trials challenging. As a result of the rarity of the disease, mitochondrial care networks for patients are needed to coordinate patient care nationally and internationally [47].

The pathophysiology of many mitochondrial diseases, such as MELAS and mitochondrial cardiomyopathies of varying presentation remains poorly characterized. The ability to achieve accurate and descriptive diagnostic criteria and identify therapeutic strategies for mitochondrial diseases likely lies in successfully modeling mitochondrial disease at the cellular level utilizing human cells.

### 4.1. Diagnosis of Mitochondrial Disease and the Use of Primary Human Tissues

Diagnosis of mitochondrial diseases remains an enormous challenge for clinicians and researchers. Although new biomarkers are being discovered that differentiate patients with mitochondrial disease from referents [3], progress is slow. Currently, when a mitochondrial disease is suspected, a blood sample is evaluated for possible causal mitochondrial variants; however, mitochondrial variants in the blood are often selected against as a result of high cellular turnover rates. Consequently, biopsy of other tissues, such as urinary epithelial cells, buccal mucosal cells, and skeletal muscle is often required for diagnosis [30]. Skeletal muscle biopsies are the current mainstay of diagnosis for most mitochondrial diseases as skeletal muscle reflects a post-mitotic tissue, enabling accurate sampling and detection of heteroplasmic casual variants compared to blood, and can be more readily accessed [48].

Primary human tissue biopsies from individuals with a given mitochondrial disease remain the most suitable for direct assessment of the biochemical and histological components of the pathophysiology. Tissue obtained directly from an individual with the mitochondrial disease myoclonic epilepsy with ragged red fibers (MERRF), for example, provides histologic evidence that rules out all other pathologies [49]. Often, however, the affected sites in patients with a mitochondrial disease, particularly those with multisystemic disease, are inaccessible. Additionally, many mitochondrial diseases present in tissues that are rarely accessible and/or unsafe to biopsy routinely, such as the heart, brain, and liver. Further, biopsies may not even contain defective mitochondrial samples due to somatic mosaicism. Importantly, primary tissue cannot provide any insight into the causality of any one factor, providing only correlation between genetic variation, histopathology, and clinical phenotypes.

### 4.2. Benefits and Drawbacks of Modeling Mitochondrial Disease in Animals

As primary tissues cannot provide causality, scientists often turn to animal models in which they can experimentally modulate exposures and, hopefully, isolate the etiology of a given disease. Animal studies have revealed many mechanisms whereby the mutation of a given OXPHOS subunit, or another component of mitochondrial function, results in disease [50]. These mechanistic studies have suggested the potential for several therapeutic targets in mitochondrial disease [51,52,53,54]. Additionally, animal models have allowed scientists to probe the causal relationship between variant introduction and cellular pathophysiology and histopathology. The drawback, however, lies in the differences in mitochondrial genetic variation between humans and animals.

The mitochondrial and nuclear genomes in eukaryotes have co-evolved, which has led to a divergence in mitochondrial function, corresponding to the divergence in eukaryotic species. Interspecies divergence has been illustrated in studies where human cells received mitochondrial transplants from the very closely related chimpanzee or gorilla. The inter-species mitochondrial transfer revealed an incompatibility of nuclear-derived mitochondrial OXPHOS subunits with mitochondrial-derived OXPHOS subunits and the cells displayed deficiencies in OXPHOS [55]. Similar studies exchanging mitochondria (and their genomes) between rat and mouse cells revealed a similar incompatibility [56], highlighting the potential lack of translatability of most mtDNA studies from animal models to humans. Because some mitochondrial genetic loci are conserved across the majority of animals, some animal studies have demonstrated the consequences of mitochondrial genetic perturbation in a way applicable to humans. While animal models of mitochondrial disease caused by homogenic, highly conserved mutations are currently being employed to study human mitochondrial disease [57], the utility in understanding the pathobiology of many of the causal mutations in mitochondrial disease has been hindered by the inter-species differences between human mtDNA-nDNA interactions and those of other animals. Consequently, validating and determining the translatability of the underlying mechanisms of mtDNA mutations in human cells remains of critical importance.

### 4.3. Disease Modeling in Human Induced-Pluripotent Stem Cell-Derived Mature Cells

The differences between animals and humans have raised the question: how do we effectively study mitochondrial mutations of unknown significance in a human genetic background on cell function? Historically, cybrids have been generated using human cell lines depleted of their mtDNA with ethidium bromide and repopulated with mitochondria through the fusion of an enucleated cell containing the mtDNA of interest [55,58] Cybrid cell lines have provided insights into the contribution of mitochondrial genetic variants to cellular phenotypes; however, the cell types utilized to generate cybrids are restricted to cancer cell lines, typically osteosarcoma cells, which already have an abnormal metabolic phenotype, very different from that of non-cancerous cells [55,58,59]. Hence, human iPSCs, with patient-specific genetic background, and the ability to use CRISPR-Cas gene editing to introduce specific mutations, have the potential to greatly advance our ability to study mitochondrial genetic variants on cellular phenotypes.

In contrast to primary human tissues, which are difficult to obtain, human iPSCs are readily obtained through the genetic reprogramming of either skin fibroblasts or peripheral blood mononuclear cells [60,61]. Following the ethical debates surrounding the use of embryonic stem cells, and the search for more readily available sources of similar pluripotent stem cells, Dr. Shinya Yamanaka’s laboratory identified four transcription factors that induce the reprogramming of somatic cells into pluripotent cells that exhibit a functional and molecular phenotype highly similar to embryonic stem cells. Since the resulting cells were derived through reprogramming Yamanaka and colleagues named these cells “induced pluripotent stem cells” (iPSCs). As with embryonic stem cells, iPSCs are pluripotent as they can be differentiated in vitro into any adult cell lineage of all three germ layers, typically through the approach known as “directed differentiation”. Directed differentiation is the process whereby sequential culture conditions are applied to stimulate a sequence of cell fate decisions in vitro that recapitulate the series of milestones known to occur during the in vivo embryonic development of that cell type [60].

The advent of iPSC technology revolutionized medicine, as iPSCs’ derivation from patient somatic cells, along with the optimization of culture conditions for their directed differentiation, now provides an inexhaustible source of patient-specific cells that could be differentiated in a targeted way towards any adult cell type. Importantly, iPSCs possess the unique genetic background of a given patient, enabling research into the precise mechanism of disease for that individual. Moreover, iPSC-derived cells allow targeted drug screens to be performed using cells that carry a given patient’s genetic background, enabling facile selection of already FDA-approved therapeutics for the management of their illness, as well as the screening of safety and efficacy of novel drug candidates.

Several labs have already begun to utilize iPSC technology to model human mitochondrial disease [62,63,64,65,66,67]. Until recently, gene-editing tools were unable to be targeted into the mitochondria making it challenging to edit the mitochondrial genome. Hence, iPSCs created from patients with a suspected or confirmed mitochondrial disease are potentially effective for modeling mitochondrial genetic diseases in cases where the disease results from nDNA mutations, or when diseased mitochondria carrying mutant mtDNA are present in the cytoplasm of the starting reprogrammed cell from which an iPSC line is clonally derived. Thus far, several groups have utilized iPSCs to model MELAS-like neurons [62,63]. Other groups have derived iPSCs for the purpose of determining how mitochondrial genetic variants present in the patient segregate in culture [65,68], and how the mitochondrial variants affect the directed differentiation process into committed adult phenotypes [62]. Few studies, however, have made use of iPSC and differentiation technologies to characterize the physiological effects of mitochondrial variants of unknown or suspected pathological significance in differentiated cells.

The large promise, in our view, of patient-derived iPSCs is in the ability to study the biochemical and subcellular pathophysiology of mitochondrial variants suspected but not confirmed to contribute to disease. As previously stated, many mitochondrial variants are correlated with multisystem disease phenotypes, but no treatment options are available as the molecular signaling pathways, transcriptional and metabolic states, and epigenomic changes that result from perturbed mitochondrial function in an organ-specific manner have yet to be characterized. iPSCs harboring mtDNA variants stand to play a significant role in allowing scientists to identify how mitochondrial variation affects mitochondrial and cellular function.

### 4.4. Targeted Gene-Editing and Its Use in Identifying Functionally Relevant Variants

In addition to the use of human iPSC lines to gain mechanistic insight into cellular disease processes, the recent discovery of *CRISPR/Cas9* gene-editing technology has quickly changed the way we understand genetic variation and disease [69,70,71]. Previous gene-editing techniques, including the Cre-lox recombinase mechanisms of rearrangement, were complex and cumbersome requiring the introduction of additional sequences and exogenous pharmacological compounds to activate the recombination mechanism. Earlier gene-editing technologies such as zinc finger nucleases and TALENs utilized multicomponent editing systems that relied on induction of a locus-specific DNA double strand break, paving the way for subsequent *Cas9*-based editing. *CRISPR-Cas9* obviated the need for anything except a guide RNA to facilitate targeted binding, the *Cas9* protein for creating a double stranded DNA break, and, if correcting via homology-directed repair, a template sequence to introduce the variant of interest. While this system is still being optimized to act site-specifically and efficiently to generate homozygous bi-allelic edits without off target DNA insertions or deletions, it is highly promising for use in understanding human genetic variation and as a gene therapy.

Scientists now possess the ability to introduce site-specific mutations into the nuclear genome [69,72,73] and even correct mutations to demonstrate the causal role of a specific mutation in the disease process. As such, *CRISPR-Cas9* technology has quickly been coupled with iPSCs to study disease-causing genetic variation in humans. Until recently, targeting of the mtDNA with *CRISPR-Cas9* was unfeasible due to challenges in targeting the guide RNA and Cas9 protein to the mitochondrial matrix [74,75,76]. Consequently, most studies to-date have utilized *CRISPR-Cas9* technologies to edit nDNA-encoded mitochondrial genes. A large advantage of *CRISPR-Cas9* editing in studying mitochondrial genetic variants in particular, remains that one can introduce mutations of interest that may be difficult to obtain from patient samples, and we are not limited by the geographic location of a patient with a specific disease-causing variant.

While mtDNA gene editing using *CRISPR/Cas9* remains more difficult than nuclear editing, recent studies have demonstrated success doing so. In updating the method for targeting the highly inaccessible mitochondrial matrix where the mtDNA is housed, scientists have shown that by combining (1) an endogenously expressed *Cas9* with a mitochondrial targeting sequence with (2) an NADH:ubiquinone oxidoreductase chain 4 targeting guide RNA, in conjunction with an RNA transport-derived stem loop element, the mtDNA can be edited [76]. In addition to advances in *CRISPR/Cas9*, a non-*CRISPR*-based approach utilizing bacterial cytidine deaminase toxin demonstrated efficiency in catalyzing C•G-to-T•A conversions within the mtDNA with high target specificity. This alternative approach has the added benefit of not requiring endogenously encoded Cas9 proteins, which must be inserted into the nDNA, and yields functional changes in metabolism when targeting mitochondrially-encoded respiratory genes [77].

Targeted gene editing, in either the nuclear or mitochondrial genome, facilitates an enhanced ability with iPSCs to understand variants of unknown significance, as correction of these mutations will likely reveal their individual contributions, however large or small, to the disease phenotype at the cellular level. Additionally, targeted gene editing facilitates interrogation of nDNA genes suspected to interact with mtDNA genes, and identification of how variants interact with different mitochondrial haplogroups. Lastly, the inefficiency of bi-allelic reintroductions of genes of interest using *CRISPR-Cas9* is advantageous, as one can introduce heterozygous nDNA mutations and create heteroplasmic mitochondrial populations. Importantly, the heterozygosity derived from *CRISPR-Cas9*-mediated gene editing will be advantageous in identifying the biochemical thresholds for disease across different mitochondrial mutations, as homozygous mitochondrial mutants in the nDNA and homoplasmic pathogenic mtDNA variants usually result in cell death. Altogether, these studies have shown potential for the use of CRISPR-Cas9 edited human iPSC-derived adult cells in delineating the mechanisms responsible for mitochondrial dysfunction in genetic diseases.

## 5. iPSCs and -Omics in Disease Modeling: Revealing Mitochondrial Mechanisms 

In addition to iPSC models, the application of large-scale -omics (including epigenomics, transcriptomics, proteomics, and metabolomics) has aided in more completely characterizing mitochondrial diseases. At the whole organism level, -omics approaches such as metabolomics have revealed unifying signatures of mitochondrial diseases, as previously discussed. The utility of -omics in mitochondrial disease is a great, as the potential for identifying biomarkers is greatly enhanced. iPSCs, which facilitate the creation of new models of human disease, stand to play a significant role in helping model the effects of mtDNA variation on metabolism and cellular function. Moreover, single-cell level -omics in conjunction with iPSC models of human mitochondrial genetic variation stand to play a significant role in identifying the cellular pathobiological mechanisms of mitochondrial disease. Single cell -omics will help reveal how heteroplasmic variants along a sliding scale of percentages of the mtDNA population in a given cell contribute to the disease phenotype and have limitless potential when considered across all genes and in multiple cell types.

### 5.1. Mitochondrial Cardiomyopathy: iPSC Models, Mechanisms, and Novel Therapeutic Targets

**Figure 3:**
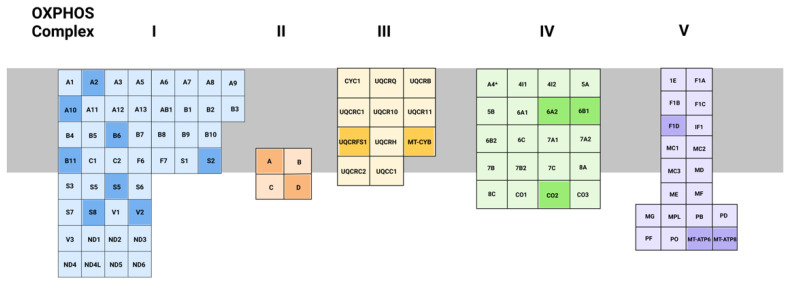
Illustration of the oxidative phosphorylation complexes and their respective subunits. Subunits are listed in alphabetical order in each complex and as such, complex prefixes have been left off. For example, in complex I, prefix NDU-; complex II SDH-; etc. Subunits shaded darker are reflective of genes with mutations curated from Origin of Mendelian Inheritance in Man (OMIM) by performing a search for “mitochondrial cardiomyopathy” on 4 January 2020.

Mitochondrial cardiomyopathies have varying etiologies and clinical presentations, with the mechanisms remaining undiscovered. Mutations in mitochondrial genes in both mtDNA and nDNA can result in mitochondrial cardiomyopathies (Table 1 and Figure 3) [78]. The use of iPSC-derived cardiomyocyte models, in conjunction with CRISPR-Cas9 gene editing, now makes it possible to model mitochondrial cardiomyopathy and study the cellular pathobiology of mitochondrial cardiomyopathy [79,80]. Several studies have demonstrated the ability of iPSC-derived cardiomyocytes to recapitulate various cardiomyopathy phenotypes such as hypertrophy, dilation, and metabolic dysfunction [80,81,82,83], but such methodologies have yet to be utilized to systematically, and comprehensively, evaluate mutations that cause mitochondrial cardiomyopathy.

One study has demonstrated the utility of using iPSC-derived cardiomyocytes for screening novel therapeutic strategies in mitochondrial cardiomyopathy. iPSC-derived cardiomyocytes were generated from pediatric patients carrying a loss of function mutation in the *DNAJC19* gene (rs137854888). The iPSC-derived cardiomyocytes carrying the *DNAJC19* gene mutation had differential levels of cardiolipin species and mitochondrial fragmentation, pathophysiological signs previously associated with dilated cardiomyopathy with ataxia (DCMA), a phenotype often diagnosed with Barth syndrome. Treatment with SS-31 in part restored mitochondrial networks [84], although the mechanism, which appears to be linked to mitochondrial membrane lipid cardiolipin, remains unclear. While this study did not use *CRISPR-Cas9* editing, it does reveal the utility of human iPSC-derived cardiomyocytes harboring mitochondrial genetic variants in studying the mechanisms of mitochondrial disease. As aforementioned, the additional application of CRISPR-Cas9 will allow scientists to effectively model variants that are difficult to obtain from patient populations; for example by editing normal iPSCs to introduce variants of uncertain significance that have been hypothesized to result in mitochondrial dysfunction. Importantly, for mitochondrial mutant models that do exist from patients, many have yet to be phenotypically validated [85]. This may mean that extensive validation of iPSC-derived lines will be needed to ensure accurate recapitulation of clinical phenotypes.

### 5.2. Mitochondrial Disease Modeling across Different Cell Types

While mitochondrial cardiomyopathy only represents one clinical presentation of mitochondrial mutations, the opportunities for modeling mitochondrial disease are ex-pansive. Just as many iPSC models of nDNA mutants have been created to model human disease [73,86,87,88] we postulate that the advancements in mtDNA gene editing will facilitate a wide breadth of mitochondrial disease modeling. With the ability of iPSCs to undergo directed differentiation towards any adult cell type, it is now possible to systematically evaluate the effects of mitochondrial genetic variation on cellular phenotype. To-date, most studies of mitochondrial genetic variation have only used undifferentiated iPSCs, or have differentiated the iPSCs into a select few cell types including neurons/neural progenitor cells, fibroblasts, and cardiomyocytes [52,56,64,68,73]. iPSCs have been generated from patients harboring variants that cause mitochondrial disease, illustrating the feasibility of creating patient-derived unique cell lines [74] However, the limit in breadth of cell type differentiation, the small selection of mitochondrial genes studied, and the lack of investigation of how differing heteroplasmic variant levels affect cellular phenotype have limited the realization of the full potential of iPSCs and CRISPR/Cas9 gene-editing techniques in advancing our understanding of mitochondrial genetic variation in disease.

## 6. Limitations and Room for Optimization in iPSC Modeling

Despite the advantages conferred over animal models and primary human tissues, iPSCs have several limitations in their ability to model mitochondrial disease. First, while iPSCs possess the ability to differentiate into any cell type, some mitochondrial mutations disrupt the differentiation process, particularly directed differentiation toward highly energetic cell types [62]. If a given mitochondrial genotype severely impairs respiratory chain function, the differentiation to highly energetic cell types may not be feasible. As such, not all mitochondrial genetic variants of interest will be able to be successfully modeled in iPSC-derived adult cells. Additionally, a recent report described the effect of reprogramming of fibroblasts to iPSCs on the presence of heteroplasmic mutations, and suggested that age-acquired mtDNA variants do not persist through reprogramming. This may mean that only inherited mtDNA heteroplasmic variants in patient-derived iPSCs can be modeled without using gene editing [89]. This report did find, however, that the heteroplasmic variants present in the iPSCs are not lost and remain at similar levels in the subclones, suggesting that the heteroplasmic variants present in an iPSC line are stable. Together, these studies highlight the importance of genetic sequencing in validating genotypes at multiple time points in a given study.

In addition to the limitations in genetic modeling and differentiation, iPSCs that are successfully differentiated do not always possess the characteristics of the fully-mature adult cell type, retaining a more fetal-like phenotype. For example, while iPSC-derived cardiomyocytes contract and express cardiac muscle-specific genes, they lack adult metabolic phenotypes and calcium handling properties [90,91,92]. Interestingly, emerging studies have demonstrated that modulation of the metabolic components provided in the media enhances the maturation of iPSC-derived cardiomyocytes [93,94]. Currently, multiple strategies are being evaluated for improving the maturation of iPSC-derived cells to ensure translatable findings, including optimization of media conditions and alternative culture substrates [93,95,96]. Lastly, with varying efficiencies of differentiation and heterogeneity of iPSC-derived populations, cultures can contain multiple cell types, not all of which are of interest. Despite the limitations in iPSC technologies, with continued advancement, it holds a great deal of promise in advancing our understanding of rare mitochondrial diseases, which will likely inform us of more common diseases with a mitochondrial component.

## 7. Conclusions

**Figure 4:**
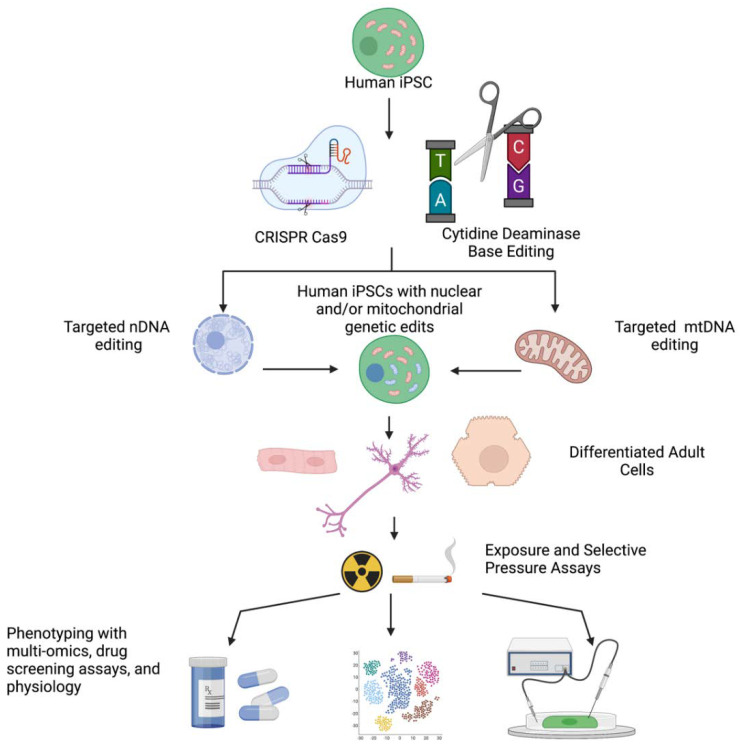
iPSCs edited with CRISPR-Cas9 (nDNA) and cytidine deaminase base editing (mtDNA) can be utilized for the identification of novel mechanisms and treatments in mitochondrial diseases. Figures created with biorender.com (accessed on 10 October 2021).

Mitochondrial disease pathobiology remains incompletely understood due to the absence of disease models. We posit that the recent advancements in human iPSC technology, in conjunction with CRISPR-Cas9 targeted gene-editing techniques, will significantly extend our ability to systematically investigate the effect of individual or combinations of nDNA or mtDNA-encoded mitochondrial gene mutations, on mitochondrial function in various adult cell types. The use of multi-omics approaches, particularly at the single cell level in iPSCs, positions the iPSC to be an effective model system for mitochondrial metabolic disorders. Moreover, the ease of iPSC-based screening assays for environment-gene interactions will enhance our understanding of the effect of these interactions on cell phenotypes. Advancement in our understanding of mitochondrial pathobiology will aid in the identification of therapeutic targets in the management of what are currently difficult diseases to treat. Our enhanced understanding will enable drug screening assays that include patient-specific human genetic backgrounds in adult cell types and are likely to identify novel treatment strategies (Figure 4).

## Figures and Tables

**Table 1:** nDNA and mtDNA mutations resulting in mitochondrial cardiomyopathies.

Gene	Protein	Mutation
*TKFC*	Triokinase and FMN cyclase	1628G > T
*FLAD1*	Flavin adenine dinucleotide synthetase 1	526_537delinsCA
*NDUFS2*	NADH:ubiquinone oxidoreductase core subunit S2	683G > A, 686C > A, 1237T > C
*NDUFA2*	NADH:ubiquinone oxidoreductase subunit A2	IVS2DS, G-A, +5
*NDUFB11*	NADH dehydrogenase [ubiquinone] 1 beta subcomplex subunit 11	IVS1DS, G-A, +5, 262C > T, 402delG
*NDUFS4*	NADH:ubiquinone oxidoreductase subunit S4	44G˃A, 316C > T
*NDUFS8*	NADH:ubiquinone oxidoreductase core subunit S8	236C > T, 305G > A, 229C > T, 476C > A
*NDUFA10*	NADH:ubiquinone oxidoreductase subunit A10	1A > G, 425A > G
*NDUFV2*	NADH:ubiquinone oxidoreductase core subunit V2	IVS2+5_+8delGTTA, 669_670insG
*SDHA*	Succinate dehydrogenase complexf lavoprotein subunit A	1664G > A
*SDHD*	Succinate dehydrogenase complex subunit D	275A > G
*COQ4*	Coenzyme Q4	433C > G, 421C > T, 718C > T, 202G > C
*UQCRFS1*	Ubiquinol-cytochrome c reductase, Rieske iron-sulfur polypeptide 1	610C > T
*MT-CYB*	Mitochondrially-encoded cytochrome b	15498 G˃A
*MT-CO2*	Mitochondrially-encoded cytochrome c oxidase subunit 2	7896G > A
*COX6A2*	Cytochrome c oxidase subunit 6A2	117C > A
*COX6B1*	Cytochrome c oxidase subunit 6B1	58C > T
*COX10*	Cytochrome c oxidase subunit 10	791C > A, 1211A > T
*COX14*	Cytochrome c oxidase subunit 14	88G > A
*COX15*	Cytochrome c oxidase subunit 15	700C > T
*ATP5F1D*	ATP synthase F1 subunit delta	245C > T
*MT-ATP6*	ATP synthase F0 subunit 6	8993T˃G, 8528T > C
*MT-ATP8*	ATP synthase F0 subunit 8	8528 T˃C
*MC5DN6*	ATP synthase membrane subunit DAPIT	87+1G > C, +1
*PPA2*	Inorganic pyrophosphatase 2	280A˃G, 318G > T, 380C > T, 500C > T, 514G > A, 683C > T
